# Exposure Monitoring and Risk Assessment of Biphenyl in the Workplace

**DOI:** 10.3390/ijerph120505116

**Published:** 2015-05-13

**Authors:** Hyeon-Yeong Kim, Sae-Mi Shin, Miran Ham, Cheol-Hong Lim, Sang-Hoon Byeon

**Affiliations:** 1 Occupational Safety and Health Research Institute, Korea Occupational Safety & Health Agency, Daejeon 305-380, Korea; E-Mails: kk3843@yahoo.co.kr (H.-Y.K.); limch@kosha.net (C.-H.L.); 2 Department of Environmental Health, College of Health Science, Korea University, Seoul 136-701, Korea; E-Mails: saemishin@naver.com (S.-M.S.); miran5072@naver.com (M.H.)

**Keywords:** biphenyl, response, exposure, industrial, toxicity, carcinogen

## Abstract

This study was performed to assess exposure to and the risk caused by biphenyl in the workplace. Biphenyl is widely used as a heat transfer medium and as an emulsifier and polish in industry. Vapor or high levels of dust inhalation and dermal exposure to biphenyl can cause eye inflammation, irritation of respiratory organs, and permanent lesions in the liver and nervous system. In this study, the workplace environment concentrations were assessed as central tendency exposure and reasonable maximum exposure and were shown to be 0.03 and 0.12 mg/m^3^, respectively. In addition, the carcinogenic risk of biphenyl as determined by risk assessment was 0.14 × 10^−4^ (central tendency exposure) and 0.56 × 10^−4^ (reasonable maximum exposure), which is below the acceptable risk value of 1.0 × 10^−4^. Furthermore, the central tendency exposure and reasonable maximum exposure hazard quotients were 0.01 and 0.06 for oral toxicity, 0.05 and 0.23 for inhalation toxicity, and 0.08 and 0.39 for reproduction toxicity, respectively, which are all lower than the acceptable hazard quotient of 1.0. Therefore, exposure to biphenyl was found to be safe in current workplace environments. Because occupational exposure limits are based on socioeconomic assessment, they are generally higher than true values seen in toxicity experiments. Based on the results of exposure monitoring of biphenyl, the current occupational exposure limits in Korea could be reviewed.

## 1. Introduction

Biphenyl is used as an intermediate agent such as an emulsifier, polish, and protectant in various production activities. Because of its high thermal stability, biphenyl oxide alone or as a mixture is used as a heat transfer material [1]. Biphenyl is naturally generated in coal tar, stock, and natural gas. In South Korea, the production and handling of biphenyl occurs in 26 locations and yields approximately 30,000 tons/year. Biphenyl is used as a high production volume material in 2220 places in the United States, yielding approximately 16,000 tons/year [2].

Biphenyl is converted to hydroxyl derivatives in the rat after ingestion. Urine specimens that were collected after rats consumed a synthetic diet with 1% biphenyl contained the following: 4-hydroxybiphenyl (30%), biphenyl glucuronide (18.4%), 4,4’-dihydroxybiphenyl (5.3%), 3,4-dihydroxybiphenyl (3.1%), and diphenylmercapturic acid (1.3%) [1]. Although effect for human was not proved, biphenyl had carcinogenicity and reproductive toxicity to animal experiments [3,4,5].

Vapor or high levels of dust inhalation and dermal exposure to biphenyl can cause eye inflammation, irritation of respiratory organs, and permanent lesions in the liver and nervous system [2,6]. In one report, workers in France who were exposed to biphenyl vapor developed vomiting and bronchitis [1]. In a study in Finland in 1969, exposure to biphenyl for 11 years led to the death of workers, which resulted in a large investigation into the effects of the paper-making process on workers [7]. The clinical symptoms of biphenyl poisoning include damage to the central and peripheral nervous systems, whereas chronic exposure is characterized by headache, fatigue, insomnia, and sensory impairment, often accompanied by cardiac or hepatic impairment [2,6,8,9]. According to the material safety data sheet from the Korea Occupational Safety & Health Agency, biphenyl is classified as category 1B (suspected carcinogen for human) because it is a carcinogen, category 3 (respiratory irritation or anesthesia for human) because of its specific target organ toxicity (single exposure), and category 1 (severe toxic for human by repeated exposure) [10]. The occupational exposure limit (OEL) of biphenyl in Korea is 0.2 ppm (1.5 mg/m^3^), the same as the threshold exposure limit established by the American Conference of Governmental Industrial Hygienists, which was determined based on experimental results using mice that showed difficulty breathing at 1 ppm (5 mg/m^3^).

Risk assessment began when the US Food and Drug Administration banned the use of food additives with the Delay Act in 1970. Four steps are required for risk assessment according to the US National Research Council: hazard identification, dose-response assessment, exposure assessment, and risk characterization [11,12].

In the European Union, hazards risk assessment technical guidelines are provided for all new chemicals and to regulate existing chemical guidelines [13]. In recent years, Registration, Evaluation, Authorization, and Restriction of Chemicals in the European Union has aimed to apply these guidelines to companies. In Japan, risk assessment has been conducted since 2006 according to the proposed method in the Risk Assessment Review Report for the Health Disturbance Prevention of the Worker Ministry of Health [14].

International chemical management involves evaluating the hazards and risk of high production volume chemicals and substances of very high concern. Until now, regulatory target chemical selection and regulation in Korea has been mainly based on the introduction of foreign standards more than assessing risk using internal guidelines. However, as the chemical industry in Korea grows, applying a foreign standard is no longer the best method, and local standards are needed. This is especially the case in cutting-edge industries in which Korea plays a leading role, such as electronics and nanotechnology. In such industries, a health risk assessment system is required to manage the use of chemicals. Therefore, proactive risk assessment and chemical management based on health risk assessment is needed. A risk assessment system was introduced by the Korea Ministry of Employment and Labor in 2013 based on changes in domestic industries, such as the development of electricity, electronics, and other high-tech industries [11,15]. The present study investigated the exposure level of biphenyl for workers in workplaces in which the health risk assessment was conducted. In addition, this study provided observational epidemiological data to help assess if the current OEL of biphenyl in Korea is appropriate.

## 2. Experimental Section

### 2.1. Sampling and Analytical Methods

A total of 38 personal and area samples were collected from three workplaces that use biphenyl for heat transfer in textile manufacture, in plastic manufacturing, and as the electrolyte in lithium battery manufacture. The workplaces were located in Jeonjusi, Jeonrabook-do (A, B), and Gongju-si, Chungcheongnam-do (C), respectively. Workplace A produced plastic for engineering, textiles for industrial materials, and ion exchange resins for PET bottles. The processes in workplace A were divided into manufacturing and injection molding, and biphenyl was used as heat transfer medium to maintain the temperature of raw materials. Workplace B produced the electrolyte of lithium batteries by adding salt, organic solvent, and additives at a steady rate. Ethylene carbonate, propylene carbonate, dimethyl carbonate, diethyl carbonate, and ethyl methyl carbonate were used as solvents for salt, and an additive was used to prevent overcharge, improve the conducting property, and make it flame retardant. A small amount of biphenyl was used as an additive in this process. Workplace C manufactured textiles. Biphenyl did not react and was used as a thermal insulating fluid to set the temperature for polymerization. Workers were always in the control room and watched the process; when problems occurred during the process, they maintained and repaired the issue. The probability of exposure to biphenyl was expected to be low for workers because biphenyl only moves through transfer tubes for raw materials. Workplaces A, B, and C had local exhaust ventilation systems which had worked well for each work process. They managed the material safety data sheet well and attached warnings to the containers of chemicals used in the workplace. The workers in workplaces A and B wore personal protective equipment, including half facepiece respirators with particulate filters, but the workers in workplace C did not wear that personal protective equipment. Local exhaust ventilation systems were installed in the workplace environments of the three workplaces. 

The 38 samples included 12 personal samples from workplace A, three personal samples and 11 area samples from workplace B, and six personal samples and six area samples from workplace C. Sampling and analysis were performed according to Occupational Safety and Health Administration method PV2022 [16].

Using a solid adsorbent tube (XAD-7, SKC 226-95) as the sampling media, the sampling flow rate was 0.2 L/min and total flow volume was 20 L. After desorbing the solid absorption tube using carbon disulfide, we analyzed the samples by using a gas chromatography-flame ionization detector equipped with a gas chromatography capillary column (Rtx-502.2, 30 m × 0.32 mm ID × 1.8 µm film). The analytical conditions of gas chromatography were as follows: column oven temperature was 135 °C, injector temperature was 225 °C, detector temperature was 250 °C, carrier gas was N_2_ (at 50 mL/min), and injection volume was 5 μL.

### 2.2. Uncertainty Analysis

In this study, we performed a probabilistic risk assessment to reduce the uncertainty that could be generated by applying a single value for the exposure variables. The results of the workplace environmental measurements were entered as fitting methods in a Monte Carlo simulation, which is a broad class of computational algorithms that rely on repeated random sampling to obtain numerical results; typically, simulations are run many times over to obtain the distribution of an unknown probabilistic entity, and the optimum function is induced. Monte Carlo simulation was performed 100,000 times for each simulation input using Crystal Ball (Oracle, Denver, CO, USA, version 11.11). As a result, the central tendency exposure (CTE) (median) and reasonable maximum exposure (RME) (cumulative 95%) values of the exposure concentration were calculated. CTE is a central and typical value for a probability distribution that can be calculated for either a finite set of values or a theoretical distribution, such as the normal distribution. In this study, the form is the geometric mean, which is the common value measured in exposure assessment. RME means typically the 95% upper confidence limit of the toxicant distribution. 

### 2.3. Risk Assessment

Risk assessment is performed by four steps: hazard identification, dose-response assessment, exposure assessment, and risk characterization. Each step is performed as follows.

### 2.4. Hazard Identification and Dose-Response Assessment

Hazard identification is conducted by qualitatively and quantitatively analyzing the health effects of chemicals through research. The hazard of biphenyl is broadly identified, so this study used existing databases such as the OECD Screening Information Data Set, the European Chemicals Bureau Report, the European Chemicals Agency Annex XV Report, and the US Environmental Protection Agency (EPA) Integrated Risk Information System Report.

Dose-response assessment is performed to determine the amount of risk based on the hazard of chemicals. This step has to be conducted separately for carcinogenic toxicity and non-carcinogenic toxicity because they have different features and hazards; carcinogen chemicals have no threshold, but non-carcinogen chemicals have a threshold. Biphenyl has carcinogenic toxicity and non-carcinogenic toxicity, so it was necessary to perform this assessment for both. 

Carcinogen assessment. Carcinogen assessment involves obtaining quantified information to determine the minimum carcinogenesis-affecting risk values from an experimental animal or the relative danger of cancer obtained from an epidemiological survey of the person. By using the smallest carcinogenesis-affecting value obtained mainly from animal experiments, quantified values can be provided as the inhalation unit risk or oral slope factor according to the US EPA. 

Dose-response assessments are conducted in many countries and organizations using these values [11]. To obtain the unit risk at workplace (UR_work_) value, the values presented were divided by the correction factor. The correction factor was determined by considering worker breathing volume (10 m^3^/day), worker exposure day (260 days), exposure period (40 years), and weight (60 kg).

Non-carcinogen assessment. The non-carcinogenic toxicity of biphenyl was evaluated as the reproduction toxicity and specific target organ toxicity (divided into oral and inhalation toxicities). The non-carcinogenic dose-response evaluation involves determining the no-effect concentration by using the no observed adverse effect level and lowest observed adverse effect level or the 95th percentile lower confidence limit of the benchmark dose (BMDL). To determine this value, quantified information provided by several agencies can be used; the reference concentration (RfC) or reference dose is provided by the US EPA, and the derived no effect level is provided by the European Chemicals Agency of the European Union. However, when applying the correction factor to each institution, as shown in Table 1, a significant difference is noted. Therefore, the Korea Occupational Safety & Health Agency has presented correction factors suitable for Korean workers by referring to the method of the US EPA, the European Chemicals Agency of the European Union, and the Ministry of Health, Labor and Welfare of Japan (Table 1).

### 2.5. Exposure Assessment

To obtain the concentrations for CTE and RME, the workplace environment measurement data fitting method of Monte Carlo simulation was used. Using this simulation, the optimal function was induced and the best-fit distribution was the log-logistic distribution. The calculated CTE and RME concentrations were used to derive the excess cancer risk (ECR) and hazard quotient (HQ) in the last step (risk characterization).

### 2.6. Risk Characterization

The dose-response assessment generates a value that can be used to calculate risk. If the value measured in the workplace environment is divided into the calculated UR_work_ or RfC_work_, the individual risk values for the target material can be calculated as follows:

ECR = workplace exposure concentration (mg/m^3^) × UR_work_ (per mg/m^3^) for carcinogens

HQ = workplace exposure concentration (mg/m^3^)/RfC_work_ (mg/m^3^) for non-carcinogens

**Table 1:** Correction factors for deriving the reference concentration for work.

Steps	Categories	KOSHA (Korea)	EPA (United States)	ECHA (European Union)	MHLW (Japan)
Quantitative correction	NOAEL_ADJ_	0.5	0.17	0.5	0.5
	NOAEL_HEC_	1	1	1	1
Uncertain correction	Interspecies	3	3	2.5	10
	Intraspecies	5	10	5	1
	Duration	2	3	2	1
	Severity	1	1	1	1
	Quality	1	1	1	1

KOSHA: Korea Occupational Safety & Health Agency; EPA: Environmental Protection Agency; ECHA: European Chemicals Agency; MHLW: Ministry of Health, Labour and Welfare; NOAEL_ADJ_: no observed adverse effect level of the adjusted for duration; NOAEL_HEC_: no observed adverse effect level of the adjusted to a human equivalent concentration.

## 3. Results and Discussion

### 3.1. Dose-Response Assessment

*Carcinogen toxicity.* The slope factor produced from data on the incidence of hepatic cancer in BDF1 female mice exposed to biphenyl for 2 years was 8.2 × 10^−3^ per mg/kg-day, as shown in Table 2 [4,17]. By applying a respiratory rate of 20 m^3^/day and an American mean weight of 70 kg, the inhalation unit risk was calculated as 2.3 × 10^−3^ per mg/kg-day. Therefore, the UR_work_ is 4.7 × 10^−4^ per mg/kg-day when calculated using the correction factor (Table 2), which yielded a value of 4.2, considering the breathing volume of a Korean worker, exposure day, and exposure years.

**Table 2:** Unit risk for carcinogenic dose-response assessment of biphenyl.

Steps	Categories	Values (per mg/kg-day)
Slope factor	BMDL	8.2 × 10^−3^
IUR	IUR = (SF × IR)/BW	2.3 × 10^−3^
UR_work_	UR_work_ = IUR/CF^a^	4.7 × 10^−4^

Notes: BMDL: lower confidence limit of the benchmark dose; IUR: inhalation unit risk; SF: slope factor; IR (inspirable rate): 20 m^3^/day; BW: body weight (mean weight of an American worker: 70 kg); UR_work_: unit risk in the workplace. ^a^CF (correction factor): $\left(\frac{\text{American adults 1 day inhalation rate}}{\text{Korean workers 8 hours inhalation rate}}\right)\times \left(\frac{\text{American adults annual exposure days}}{\text{Korean workers annual exposure days}}\right)\times \left(\frac{\text{American adults exposure duration}}{\text{Korean workers exposure duration}}\right)$ $=\left(\frac{20{\text{m}}^{3}}{10{\text{m}}^{3}}\right)\times \left(\frac{365\text{days}}{260\text{days}}\right)\times \left(\frac{70\text{years}}{40\text{years}}\right)=4.9$

To determine the specific target organ toxicity (oral toxicity), a BMDL of 23 mg/kg-day was selected as a point of departure (POD) in several studies [3,4,5]. This was based on two-year experiments conducted by Umeda *et al.* (2002) on non-tumoral focus of the urinary system in male and female F344 rats [5]. The RfC_work_ for oral toxicity can be calculated as shown in Table 3. First, considering both working hours and breathing rates of workers with light weights, a correction factor of 0.5 was applied as a quantitative correction. Next, correction factors of 3 and 5 were used for interspecies and intraspecies differences, respectively, as uncertainty corrections. Finally, the reference concentration in the workplace (RfC_work_) for oral toxicity was calculated as 2.13 mg/m^3^.

**Table 3:** Reference values for non-carcinogenic dose-response assessment of biphenyl

Steps	Categories			Values	
Toxicity			Special target organ toxicity (oral toxicity)	Special target organ toxicity (inhalation toxicity)	Reproductive toxicity
POD			BMDL 23 mg/kg-day (rat, oral)	LOAEL 157.75 mg/m^3^ (rat, inhalation)	BMDL 20 mg/kg-day (rat, oral)
Route-to-route extrapolation	Dose scaling from animals to humans		4	—	4
Quantitative correction	NOAEL_ADJ_		0.5	0.5	0.5
	NOAEL_HEC_		1	1	1
Uncertain correction	Interspecies		3	3	3
	Intraspecies		5	5	5
	Duration	≥4 weeks	6	6	**6**
		≥13 weeks	2	**2**	2
		≥6 months	**1**	1	1
	Severity	NOAEL	**1**	1	**1**
		LOAEL	5	**5**	5
	Quality		1	1	1
RfC_work_ (mg/m^3^)			2.13	0.53	0.31

POD: point of departure; BMDL: lower confidence limit of the benchmark dose; LOAEL: lowest observed adverse effect level; NOAEL_ADJ_: no observed adverse effect level of the adjusted for duration; NOAEL_HEC_: no observed adverse effect level of the adjusted to a human equivalent concentration; NOAEL: no observed adverse effect level; RfCwork: reference concentrations in the workplace.

In the case of inhalation toxicity, a lowest observed adverse effect level of 157.7 mg/m^3^ was selected as the POD in several studies [18–20]. This value is based on experiments performed by Sun Company Inc. (1977) using CD1 mice exposed for seven hours per day, five days per week, to vapor concentrations of 0, 157.7, and 315.3 mg/m^3^ of biphenyl for 13 weeks [20]. The RfC_work_ for inhalation toxicity can also be calculated as shown in Table 3. First, a correction factor of 4 was applied for route-to-route extrapolation because rats were used as laboratory animals in this study. Next, a quantitative correction was applied as for oral toxicity. Then, correction factors for interspecies and intraspecies differences were also applied as for oral toxicity. A factor of 2 for duration was selected because it may take approximately 13 weeks to six months to conduct this study. Finally, the RfC_work_ for oral toxicity was calculated as 0.53 mg/m^3^.

To assess reproductive toxicity, a BMDL of 20 mg/kg-day was selected as the POD in several studies [3,21,22,23]. This was based on a study by Khera *et al.* (1979) [3] using a gestation period of six to 15 weeks for Wister rat skeletal abnormalities of the fetus from a maternal oral intubation dose. The RfC_work_ for reproductive toxicity can also be calculated as shown in Table 3. First, a correction factor of 4 was applied for route-to-route extrapolation because rats were used as laboratory animals in this study. Next, the same quantitative correction as for inhalation toxicity was applied. In addition, correction factors for interspecies and intraspecies differences were applied as for inhalation toxicity. A factor of 6 for duration was selected because it may take approximately four to 13 weeks to conduct this study. Finally, the RfC_work_ for reproductive toxicity was calculated as 0.31 mg/m^3^.

### 3.2. Exposure Assessment

The mean exposure concentration for the 38 samples taken from three workplaces was 0.038 mg/m^3^. As a result of the Monte Carlo simulation, the CTE and RME concentrations were 0.03 and 0.12 mg/m^3^, respectively. 

### 3.3. Risk Characterization

As shown in Table 4, the ECRs for CTE and RME were 0.14 × 10^−4^ and 0.56 × 10^−4^, respectively. The HQs were 0.01 and 0.06 for oral toxicity, 0.05 and 0.23 for inhalation toxicity, and 0.08 and 0.39 for reproductive toxicity, respectively. However, using OELs in Korea of 1.5 mg/m^3^ (0.2 ppm), the ECR was 7.05 × 10^−4^ for carcinogen risk and the HQs were 0.7 for oral toxicity, 2.8 for inhalation toxicity, and 4.8 for reproductive toxicity (Table 5).

**Table 4:** The exposure measurement data of workplace environments.

Workplaces	N *	AM ** (mg/m^3^)	SD ^†^ (mg/m^3^)	Range (mg/m^3^)
A	12	0.034	0.048	0.001–0.145
B	14	0.014	0.022	0.001–0.057
C	12	0.066	0.075	0.001–0.240
Total	38	0.038	0.048	0.001–0.240

Notes: * the number of data points used to calculate AM and SD. ** AM: Arithmetic mean. **^†^** SD: standard deviation.

**Table 5:** Calculated risk values of biphenyl.

Categories	ECR (Carcinogenicity)	HQ (Reproductive Toxicity)	HQ (Oral Toxicity)	HQ (Inhalation Toxicity)
CTE	0.14 × 10^−4^	0.08	0.01	0.05
RME	0.56 × 10^−4^	0.39	0.06	0.23
OEL	7.05 × 10^−4^	4.8	0.7	2.8

Notes: CTE: central tendency exposure; RME: reasonable maximum exposure; OEL: occupational exposure limit; ECR: excess cancer risk; HQ: hazard quotient.

Risk assessment of the harmful effects of chemicals is essential for preventing and managing occupational disease. The administrative control of hazardous chemicals based on toxicity is possible through health risk assessments of workers exposed to the material, and such assessments provide scientific data on exposure in the workplace. Initiatives that present reasonable regulatory standards contribute to improving communication between workers, employers, and the government and encourage mutual trust.

In this study, the exposure concentrations of biphenyl for workers based on Monte Carlo simulation were 0.03 mg/m^3^ (CTE) and 0.12 mg/m^3^ (RME), respectively. Few cases of occupational biphenyl poisoning have been reported [1]. In one case, biphenyl-impregnated fruit-wrapping paper had been produced under extremely poor hygienic conditions; depending on the operation, the average biphenyl concentrations in the air ranged from 4.4 to 128 mg/m^3^ in 1959 and from 0.6 to 123 mg/m^3^ in 1970. Such exposures led to the poisoning of eight workers. The clinical features of biphenyl poisoning were characterized by central and peripheral nerve damage and liver injury, and the cause of death was acute yellow atrophy of the liver. Although the prognosis of cases of biphenyl poisoning is as yet unknown, deterioration of central and peripheral nervous system function was seen in some patients; in others, slight improvement was noted [7]. When air concentrations of biphenyl were <1 mg/m^3^, no differences in blood pressure, pulmonary function tests, serum creatinine levels, urinary protein levels, and standard blood cell counts were detectable between exposed and unexposed workers [24]. Another study examined 47 workers to determine the physical response to chronic inhalation of biphenyl. There are no symptoms such as liver and heart disorders at <1.6 ppm (1 mg/m^3^) [6]. Previous studies could be used to set the OEL value referring to exposure concentration and health effects. It is assumed that there are no adverse effects on health at exposure to this scenario in this study.

After the exposure assessment, carcinogenic and non-carcinogenic risk assessments of biphenyl were performed. Earlier experiments on chronic exposure found no clear evidence for biphenyl-induced carcinogenicity in rats [21,22,25,26], mice [27,28,29], dogs [19], or Rhesus monkeys [22]. However, these earlier studies were less informative because of various study limitations [17]. Under the Guidelines for Carcinogen Risk Assessment [30], the EPA concluded that biphenyl has “suggestive evidence of carcinogenic potential” [17] based on the study by Umeda *et al.* (2005) [4] of female BDF mice and male F344 rat livers and bladders. The unit risk of carcinogenicity, which was obtained using a slope factor of 8.2 × 10^−3^ per mg/kg-day on the basis of the experimental results of Umeda *et al.* (2005) [4], was calculated to be 4.7 × 10^−4^ per mg/kg-day.

Reproductive and developmental toxicity studies have been performed by Dow Chemical Company (1953), Ambrose *et al.* (1960), and Khera *et al.* (1979) [3,21,22]. The BMDL was found to be 20 mg/kg-day in experiments on fetal skeletal abnormalities in Wistar rats in a study by Khera *et al.* (1979) [3]. In the present study, the RfCwork was 0.31 mg/m^3^ from the oral to inhalation route-to-route extrapolation using a BMDL of 20 mg/kg-day as the POD. The EPA does not present the RfC from inhalation toxicity because the confidence interval is lower than that of oral toxicity. In a workplace environment, however, inhalation exposure takes precedence over oral exposure. Based on this and an inhalation study on the most appropriate values to use, we generated the RfCwork [11].

In a study on oral toxicity of subchronic exposure to biphenyl by Umeda *et al.* (2004) [31] using BDF1 mice, the authors showed that the no observed adverse effect level was 747 mg/kg-day, whereas in an earlier study on chronic exposure by Umeda *et al.* (2002) [5] using F344 rats, the obtained BMDL was 23 mg/kg-day by blood precipitation effects. Furthermore, a different BMDL (122 mg/kg-day) was obtained from a study by Umeda *et al.* (2005) [4] on mineral deposition in the kidney using BDF1 mice. In the present study, the RfCwork was calculated as 2.13 mg/m^3^ from the oral to inhalation route-to-route extrapolation using a BMDL of 23 mg/kg-day as the POD.

Inhalation exposure was shown in data presented by Deichmann *et al.* (1947) and Monsanto (1946) [18,19] using mice, rabbits, and rats and by Sun Company Inc. (1977) [20] using mice. Sun Company Inc. (1977) showed the results of subchronic exposure to biphenyl for 13 weeks. The lowest observed adverse effect level (157.7 mg/m^3^) from the Sun Company Inc. study was selected for deriving the RfCwork for dose-response evaluation. The RfCwork using this quantitative and uncertainty correction with repeated inhalation exposure was 0.53 mg/m^3^.

The ECRs of biphenyl when the workplace exposure concentrations were 0.03 (CTE) and 0.12 mg/m^3^ (RME) were 0.14 × 10^−4^ and 0.56 × 10^−4^, respectively. These values were less than the recommended ECR of 1 × 10^−4^ in the workplace environment; therefore, exposure was deemed relatively safe.

In addition, the HQs of oral toxicity were 0.01 (CTE) and 0.06 (RME), which are both considerably lower than the 1.0 value considered as an acceptable HQ; therefore, exposure to biphenyl was deemed safe in the current workplace exposure conditions. In terms of inhalation toxicity, the obtained HQs of 0.05 (CTE) and 0.23 (RME) are lower than the 1.0 value considered as an acceptable HQ, also showing safety in the current workplace environments. Finally, the HQs for reproductive toxicity were 0.08 (CTE) and 0.39 (RME), which are also less than 1.0.

## 4. Conclusions

The ECR for the Korean current exposure standard of 1.5 mg/m^3^ (0.2 ppm) was 7.05 × 10^−4^ (carcinogenesis) and the HQs were 0.6 (oral toxicity), 2.5 (inhalation toxicity), and 4.2 (reproductive toxicity); all but oral toxicity exceeded the acceptable ECR of 1.0 × 10^−4^ and the acceptable HQ of 1.0. Therefore, because the OEL is usually set higher than the RfCwork, workers may not be safe at these OELs. The OELs are usually determined by considering socioeconomic evaluation and technical control possibilities. Because of these characteristics of the OELs, although the exposure condition is below the exposure guidelines, it cannot be said to be at a safe level. Therefore, although the exposure rates are below the exposure limits, further investigation is needed to assess the toxicologically safe level and reduction efforts should continue until exposure is below the safe level. However, in this study, the exposure concentration of biphenyl in the workplace was below the toxicological safe level and the OEL of biphenyl was not. Although the OEL value is considered a socioeconomic effect, the OEL of biphenyl in Korea seems to be a little high, so it could be reviewed. The current OEL of biphenyl was set without a scientific rationale. Thus, studies like this are needed to scientifically set the OEL with risk assessment. This study can be used as a basis for reconsideration of the OEL value of biphenyl in Korea.
